# LTO as a Promising Anode Material for Aqueous Batteries: Synthesis Routes, Properties, and Electrode Preparation Approaches

**DOI:** 10.3390/nano16100612

**Published:** 2026-05-16

**Authors:** Maria Apostolopoulou, Emmanouil Pigounakis, Dimitra Vernardou

**Affiliations:** Department of Electrical and Computer Engineering, School of Engineering, Hellenic Mediterranean University, 71410 Heraklion, Greece; manospigounakis@gmail.com

**Keywords:** aqueous lithium-ion batteries, LTO, electrode fabrication, electrolyte engineering, WISE, interfacial stability, electrochemical performance

## Abstract

The growing penetration of renewable energy sources has intensified the demand for safe, sustainable, and cost-effective energy-storage technologies. Aqueous lithium-ion batteries are promising candidates because of their intrinsic safety and high ionic conductivity, though their deployment is limited by narrow electrochemical stability window of water. Lithium titanate oxide (LTO) has emerged as an ideal anode material for aqueous systems because of its exceptional structural stability, negligible volume change during lithiation/delithiation, and relatively high operating potential that suppresses hydrogen evolution. This review examines the peer-reviewed literature (2010–2026) on LTO-based aqueous lithium-ion batteries, focusing on the interdependence between material synthesis, electrode fabrication, electrolyte engineering, and electrochemical performance. Scalable fabrication techniques, such as spray deposition and tape casting, are discussed alongside their pact on electrode quality. Attention is given to water-in-salt, gel-polymer, and localized high-concentration electrolytes that expand the stability window and improve interfacial behavior. Overall, the review highlights how electrolyte design, electrode architecture, and processing methods can be jointly tailored to support stable and scalable LTO-based aqueous lithium-ion batteries systems.

## 1. Introduction

The global transition toward energy decarbonization has profoundly transformed electricity generation, leading to a growing reliance on renewable energy sources such as solar photovoltaics and wind power. Although these technologies offer low-carbon electricity production, their output is inherently intermittent and strongly dependent on weather conditions. This intermittency creates temporal mismatches between energy supply and demand, resulting in renewable-energy curtailment during low-demand periods and grid instability during peak consumption. Consequently, the deployment of large-scale, efficient, and safe energy-storage systems has emerged as a critical technological and economic priority for modern power grids [1,2,3]. In this framework, lithium-ion batteries continue to dominate electrochemical energy storage, with growing attention directed toward aqueous lithium-ion batteries (ALIBs) as intrinsically safer alternatives for stationary applications.

**Figure 1:**
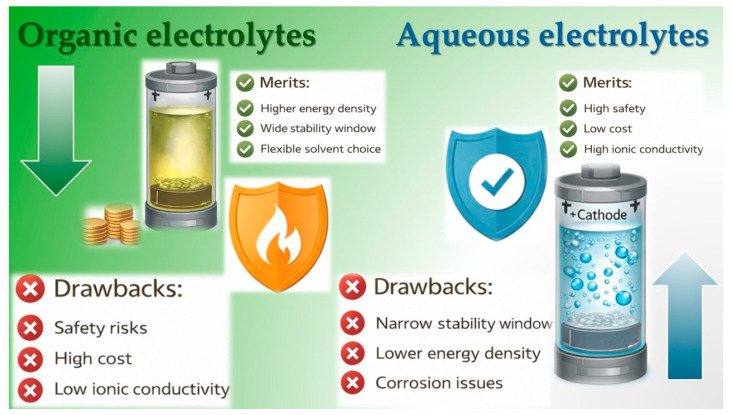
Schematic comparison between organic (non-aqueous) and aqueous electrolytes in LIBs, illustrating differences in safety, electrochemical stability window, cost, and ionic conductivity.

Figure 1 schematically compares conventional LIBs and emerging aqueous systems [4,5,6]. Organic electrolytes provide a wide electrochemical stability window and compatibility with high-energy electrode materials; however, they are associated with flammability, relatively low ionic conductivity (10^−3^–10^−4^ S·cm^−1^) [7], high cost, and environmental concerns [4,5]. In contrast, aqueous electrolytes offer intrinsic safety, higher ionic conductivity (10^−2^–10^−3^ S·cm^−1^), low cost, environmental friendliness, and simpler handling [8,9], making them attractive for stationary applications where safety and long cycle life are prioritized over maximum energy density [10].

However, the electrochemical stability window of water is thermodynamically limited by the hydrogen evolution reaction (HER) and the oxygen evolution reaction (OER) [9,10,11,12,13,14]:

At 25 °C and standard conditions, these reactions impose a theoretical electrochemical stability window of 1.23 V for water, which fundamentally limits the operating voltage of ALIBs [9,10,11,12,13,14]. This limitation has motivated the search for electrode materials capable of operating within HER-limited conditions. Among these, lithium titanate (Li_4_Ti_5_O_12_, LTO) has emerged as a particularly robust anode candidate for both aqueous and organic systems due to its zero-strain behavior (~0.2% volume change), exceptional structural stability and stable Ti^4+^/Ti^3+^ redox potential (~1.55 V vs. Li/Li^+^) that enables safe, high-rate lithium insertion.

Table 1 summarizes representative organic and aqueous lithium-ion battery systems reported in the literature. In commercial non-aqueous LIBs LTO-LFP full cell, operate at an average voltage of approximately 1.8–1.9 V and deliver specific capacities around 130 mAh g^−1^ at C/2 with negligible fading over more than 1200 cycles [15]. Also, CNTs/LTO half-cells have demonstrated specific capacities around 169 mAh g^−1^ @1 C and 112 mAh g^−1^ @20 C, [16]; however, these systems rely on flammable electrolytes and require complex safety management [3,4,5,6]. In contrast, aqueous systems employing electrode pairs such as LiMn_2_O_4_–LTO and LiFePO_4_–LTO exhibit lower operating voltages (≈1.8–1.9 V) yet deliver exceptional cycling stability, often exceeding 3000–5000 cycles with coulombic efficiencies (CEs) approaching 100% [11]. Furthermore, advanced electrolyte concepts, including water-in-salt electrolytes and gel–polymer electrolytes, have significantly expanded the accessible voltage window and narrowed the energy-density gap, while preserving the intrinsic safety and environmental compatibility of aqueous systems [11]. Overall, although ALIBs still lag conventional LIBs in terms of energy density, their combination of safety, durability, and cost-effectiveness positions them as strong candidates for stationary energy-storage applications, especially when coupled with structurally stable anodes such as LTO [11].

Recent studies on transition metal oxide (TMO) systems, such as that by Lichen Wu et al. [17], have highlighted analogous structure-interface performance interdependencies across different alkali-metal battery chemistries. Although these studies primarily focused on potassium-ion cathodes, they establish broadly applicable material design principles. Strategies such as surface engineering to enhance interfacial stability and morphology optimization to improve ion transport can be directly leveraged in the development of LTO anodes for aqueous lithium-ion systems.

Within this framework, Table 1 is not intended to provide a comprehensive comparison of battery chemistries, but rather to introduce the rationale for selecting LTO as the central focus of this review. The comparison highlights that, despite their lower operating voltage, LTO-based aqueous systems offer an advantageous combination of safety, cycling stability and structural robustness compared to many alternative configurations. Accordingly, the following sections concentrate specifically on the following parameters that govern the performance of LTO in aqueous environments: its crystal structure and zero-strain behavior, the effect of synthesis routes on particle morphology and phase purity, the role of fabrication approaches such as spray deposition and tape casting on mechanical integrity and electrochemical response, and the critical role of electrolyte engineering. Through this integrated perspective, the review highlights how LTO material properties, electrode architecture, and electrolyte design can be jointly tailored to achieve stable and scalable LTO-based aqueous LIBs.

## 2. Structural Characteristics and Synthesis Methods of LTO

### 2.1. Characteristics of LTO Structure

#### 2.1.1. Spinel Framework and Zero-Strain Mechanism

**Figure 2:**
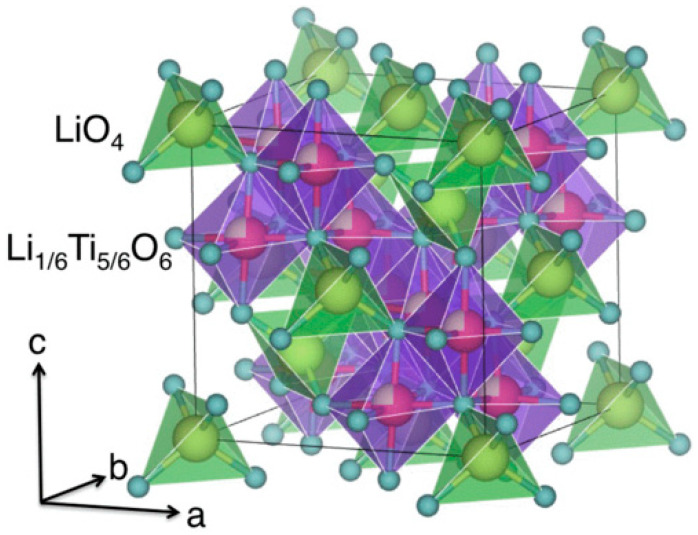
Schematic representation of the cubic spinel LTO structure. Reproduced from [21] under CC BY license.

LTO crystallizes in a cubic spinel structure (space group Fd$\bar{3}$m), in which lithium ions occupy the tetrahedral 8a sites, while Ti^4+^ ions, together with the remaining Li^+^, are arranged within the octahedral (16d sites) framework formed by oxygen atoms located at the 32e positions [14,18]. As illustrated in Figure 2, this robust spinel lattice serves as a stable host framework for lithium insertion. During lithiation, lithium ions are accommodated within the vacant octahedral 16c sites, a process that occurs with minimal volume variation (<0.2%). This remarkable structural stability is the basis of the widely accepted “zero-strain” character of LTO [19,20,21]. This near-invariant framework is directly responsible for the excellent retention of particle integrity, the preservation of electronic percolation, and the suppression of crack formation during repeated cycling compared to conventional anode materials that experience significant lattice expansion and contraction.

#### 2.1.2. Enhancing LTO Stability

Beyond its crystallographic stability, the practical performance of LTO in aqueous LIBs is strongly determined by its interfacial behavior under HER conditions. Operando and interfacial electrochemical studies indicate that HER can occur at LTO surface close to the lithiation potential in neutral aqueous electrolytes, suggesting that parasitic water reduction cannot be fully neglected under realistic operating conditions [22,23,24]. However, sustained HER activity is governed not only by thermodynamic factors [23,24,25], but also by interfacial kinetics and the catalytic properties of electrode surface, as reported by Chen et al. [26].

In this context, surface modification plays a decisive role in suppressing HER and enabling more reversible lithiation of LTO in advanced aqueous electrolytes. For instance, Al_2_O_3_ coatings have been shown to reduce HER on LTO, allowing reversible cycling in water-in-salt electrolytes with capacities of 145 mAh g^−1^. These observations demonstrate that aqueous stability of LTO depends not only on its redox potential but also on the ability of the electrode/electrolyte interface to limit catalytic water reduction [26].

**Figure 3:**
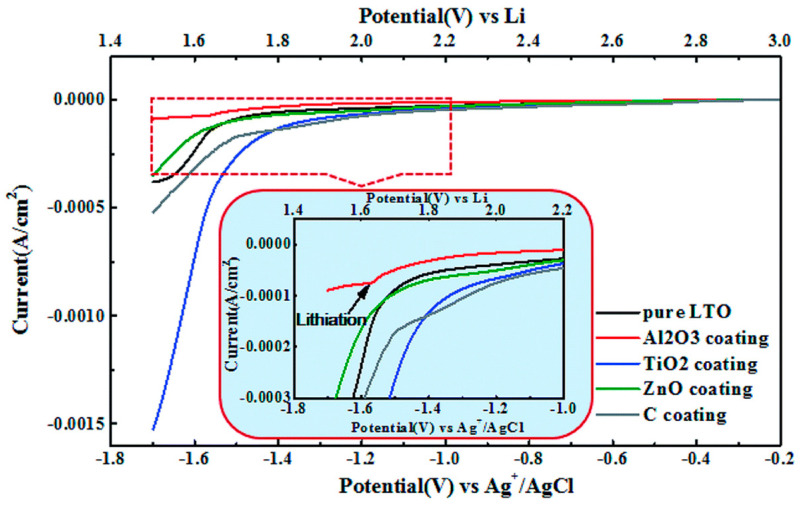
Suppression of HER at the LTO/electrolyte interface through surface modification. Reproduced with permission from [27].

As shown in Figure 3, the cathodic response of LTO is highly sensitive to surface chemistry. Compared with pristine LTO, the coated electrode exhibits a delayed HER onset and a lower cathodic current, indicating that interfacial design can effectively reduce the competition between HER and Li^+^ insertion. These findings confirm that the aqueous behavior of LTO is not dictated solely by its redox potential, but also by the catalytic and passivating characteristics of its surface. This aspect is particularly important for LTO as its zero-strain insertion mechanism suppresses crack formation and limits continuous exposure of fresh reactive surfaces during cycling. As a result, the progressive activation of HER, interfacial destabilization and impedance growth are less pronounced than in anodes that undergo substantial structural distortion or surface reconstruction [22,23,24,25,26,27]. Thus, the structural stability of LTO contributes not only to mechanical durability but also to a more stable electrochemical interface in aqueous media.

The interfacial stability of LTO can be further enhanced through electrolyte engineering. In highly concentrated aqueous electrolytes, the Li^+^ solvation environment is altered, reducing fraction of uncoordinated water molecules. This, in turn, kinetically suppresses HER and broadens the practical electrochemical stability window, as reported by Suo et al. [12] and Borodin et al. [28]. More importantly, such electrolytes can also promote the formation of a robust interphase directly on the LTO surface, further improving its electrochemical performance.

**Figure 4:**
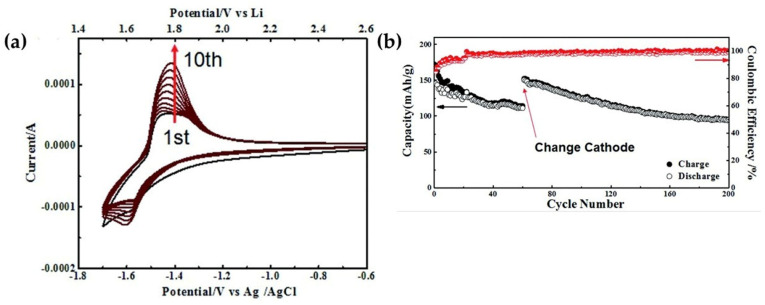
(**a**) Cyclic voltammetry of Al_2_O_3_-coated LTO vs. activated carbon in WiSE electrolyte (5 mVs^−1^). Showing the characteristic redox response and suppressed parasitic reactions, (**b**) Galvanostatic cycling performance of the LTO/LMO full cell. Reproduced with permission from [27].

Figure 4 highlights the improved electrochemical behavior of Al_2_O_3_-coated LTO in WiSE electrolytes, directly supporting the HER suppression trend identified earlier. The cyclic voltammetry profile shows a broad anodic response around 1.8 V, indicating the activation of Li^+^ diffusion following the formation of a stable SEI layer. At the same time, the onset of the cathodic HER is markedly shifted to lower potentials compared to pristine LTO, suggesting effective suppression of parasitic reactions.

This interfacial stabilization translates into improved full-cell performance. The LTO/LMO configuration delivers a stable discharge capacity of approximately 145 mAh g^−1^ (based on LTO), consistent with its theoretical limit, while maintaining high reversibility. The initial coulombic efficiency reaches 84.5%, suggesting limited electrolyte consumption due to the rapid formation of a dense, LiF-rich SEI. After this activation stage, the system stabilizes with an average CE exceeding 99% over 100 cycles and minimal capacity decay (<5%), indicating sustained interfacial integrity.

The enhanced performance is attributed to the role of the Al_2_O_3_ coating in passivating catalytically active Ti sites, enabling the formation of a compact and ionically conductive SEI that suppresses continuous electrolyte decomposition. In parallel, the reduced water activity in the WiSE electrolyte further mitigates HER, facilitating stable cycling at practical rates. Overall, the combination of surface coating and electrolyte engineering enables performance metrics comparable to advanced gel-polymer systems, underscoring its relevance for aqueous lithium-ion configurations. These surface-engineered WiSE systems establish performance benchmarks for aqueous LTO anodes.

A related stabilization pathway emerges in gel-polymer and polymer-assisted water-in-salt electrolytes, where polymer confinement reduces proton accessibility and enhances mechanical stability at the electrode/electrolyte interface. In LMO/LTO full cells, the coulombic efficiency of LTO improves dramatically from 59% (28 m WiSE, rapid decay to 37% at 5 cycles) to 93.6% (11 m polymer-assisted WiSE at 10 cycles) and 98.9% (12 m polymer-assisted WiSE), retaining ~125–158 mAhg^−1^ over 100 cycles. The 12 m solid-state aqueous polymer electrolytes achieve 99.95% CE with 142 mAh g^−1^ after 200 cycles, matching coated LTO/WiSE metrics while adding flexibility [29].

Taken together, these observations show that the aqueous stability of LTO arises from the combined effect of its intrinsically zero-strain structure and a carefully engineered electrolyte environment that minimizes interfacial water reactivity. Building on this understanding, the following sections examine electrode fabrication and electrolyte chemistry as complementary factors that determine how effectively these intrinsic structural advantages can be translated into practical aqueous battery performance. Thus, the structural stability of LTO contributes not only to mechanical durability but also to a more stable electrochemical interface in aqueous media. The following section elaborates these interfacial suppression mechanics through thermodynamic, kinetic, and solvation perspectives that underpin LTO’s aqueous compatibility.

Among the most effective surface-engineering approaches, conformal carbon coatings and atomic-layer-deposited oxide layers provide a direct means of reducing interfacial water access and suppressing catalytic decomposition on LTO. Beyond electrolyte engineering, surface modification provides a direct route to suppress interfacial water decomposition on LTO at the atomic scale. Recent LTO-specific studies have shown that conformal carbon shells synthesized by spray granulation can uniformly cover the LTO surface, reduce direct contact with the electrolyte, and suppress interfacial gas production, thereby improving cycling stability [30,31]. More broadly, carbon coating is recognized as an effective strategy to improve surface chemical stability, strengthen interfacial robustness, and facilitate lithium-ion transport, although its role is primarily related to interfacial stabilization rather than the complete elimination of HER [32]. In parallel, atomic layer deposition of metal oxides offers a more precise route to surface engineering because it provides atomic-level thickness control and excellent conformality, enabling ultrathin barrier layers that can hinder catalytic water access while preserving ion transport pathways [33,34]. Taken together, these findings indicate that HER suppression in aqueous LTO is governed not only by the intrinsic zero-strain framework, but also by the chemistry, thickness, and uniformity of the surface layer that controls water access, charge transfer, and interfacial reactivity.

### 2.2. Mechanistic Aspects of HER Suppression at LTO Anodes

Building on the surface modification effects demonstrated in Figure 3 [27], HER suppression at LTO interfaces involves district thermodynamic and kinetic contributions that distinguish LTO from graphitic or metallic anodes [22,23,27].

The standard HER potential is thermodynamically fixed at E_HER_ = 0 V_SHE_ at pH = 0, shifting to E_HER_ = −0.059 × pH V_SHE_ under neutral/alkaline conditions [21]. LTO operates at ~1.55 V vs. Li/Li^+^ (~−1.7 V vs. SHE), providing a thermodynamic overpotential margin of ~1.7 V relative to HER equilibrium [22,23]. This separation alone proves insufficient without kinetic barriers, as operando studies confirm HER onset near lithiation potentials in neural electrolytes (Figure 3) [22,23,27].

HER kinetics follow the Tafel equation n = a + b*log (i/i_0_), where i is the current density in A/cm^2^, i_0_ is exchange current density (A/cm^2^) and b is Tafel slope (120 mV/dec for Volmer-limited HER on Pt, 40 mV/dec for Heyrovsky step) [27]. LTO’s spinel TiO_2_-like surface exhibits a much lower exchange current density that Pt, owing to its unfavorable hydrogen adsorption free energy (ΔG_H_*~0.8 eV, compared with ~0 eV for Pt), thereby placing it far from the apex of the HER volcano plot [27]. The Al_2_O_3_ coatings shown in Figure 3 further elevate kinetic barriers by passivating catalytic Ti sites while preserving Li^+^ accessibility [27].

In WiSE electrolytes (>20 m LiTFSI), solvation structure reconfiguration plays a critical role. Conventional electrolytes feature Li^+^(H_2_O)_4–6_ solvation shells that facilitate proton transfer. WiSE creates anion-dominated shells (H_2_O: Li^+^ < 1), reducing free water activity and shifting HER onset from −1.6 V to −1.9 V vs. Li/Li^+^ [27]. TFSI^-^ reduction forms LiF-rich passivation layers that block water access while permitting Li^+^ diffusion (Figure 4) [27].

Together, these coupled thermodynamic, kinetic, and solvation effects transform LTO from HER-resistant into one that actively suppresses HER, enabling stable operation within expanded aqueous stability windows.

### 2.3. Synthesis Methods of LTO Powder

The synthesis of LTO is not merely a preparative step, but a key determinant of the structural and electrochemical properties that govern its behavior in aqueous LIBs. Although LTO is intrinsically characterized by its zero-strain lithiation mechanism, its practical performance depends strongly on synthesis-controlled parameters such as particle size, crystallinity, agglomeration state, phase purity and surface homogeneity [14]. These factors are particularly important in aqueous systems, where interfacial kinetics, surface reactivity and electrode–electrolyte contact often dominate the overall electrochemical response more strongly than bulk lattice effects [35,36,37].

Commercial and conventionally synthesized LTO powders often exhibit micrometer-scale particles and non-optimized microstructures, which increase Li^+^ diffusion distances and reduce the accessible active surface area. In addition, the intrinsically low electronic conductivity of LTO makes the final electrode response highly sensitive to synthesis-induced microstructural features, including particle connectivity, agglomeration and the distribution of conductive additives within the composite electrode [35,36]. For this reason, synthesis optimization should be viewed as a route not only toward phase formation but also to improve transport pathways and compatibility with practical electrode fabrication based on aqueous solutions.

Phase purity is equally critical. Minor deviations in stoichiometry or the presence of secondary phases such as anatase or rutile TiO_2_ can alter the electrochemical response and complicate interfacial stability under aqueous conditions [36,37,38]. Accordingly, the comparison of synthesis routes must account simultaneously for crystal quality, morphology control, calcination temperature, scalability and the resulting electrochemical relevance, rather than focusing on phase formation alone.

#### 2.3.1. Sol–Gel Synthesis

**Figure 5:**
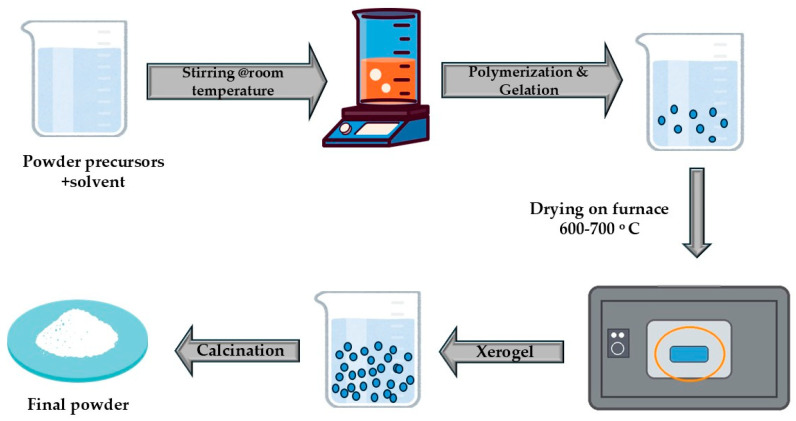
Sematic illustration of the sol–gel synthesis process.

Sol–gel synthesis is widely regarded as one of the most effective routes for producing nanoscale and compositionally homogeneous LTO. Its principal advantage lies in near-molecular-level mixing of lithium and titanium precursors before gelation, which facilitates uniform phase formation at relatively low calcination temperatures (typically 600–700 °C), as shown in Figure 5, compared with conventional solid-state processing [39,40,41].

In citrate-assisted sol–gel systems, citric acid serves both as chelating agent and carbon source, stabilizing metal-citrate complexes and suppressing premature precipitation. Wang et al. [39] demonstrated that this approach yields phase-pure LTO at 700 °C with primary particle around 500 nm [39]. Zhang et al. [40] further optimized the process using a modified citric acid method, achieving pure spinel phase at only 750 °C (vs. 850 °C in conventional synthesis) with nanocrystals of 100–200 nm resulting in 95% after 100 cycles [40]. PEG-assisted variants provide additional control over agglomeration. Zhang et al. [40] reported that polyethylene glycol templating produced uniform 200–300 nm particles with only 5% capacity fade after 500 cycles at 1 C (versus 20% PEG-free), attributed to reduced aggregation and enhanced microstructural uniformity [41]. These studies demonstrate that sol–gel methods excel in simultaneously controlling phase purity, particle size and surface homogeneity parameters that are particularly critical for LTO electrodes in ALIBs, where high interfacial areas and short Li^+^ diffusion paths improve electrochemical utilization under HER-limited conditions [39,40,41].

**Figure 6:**
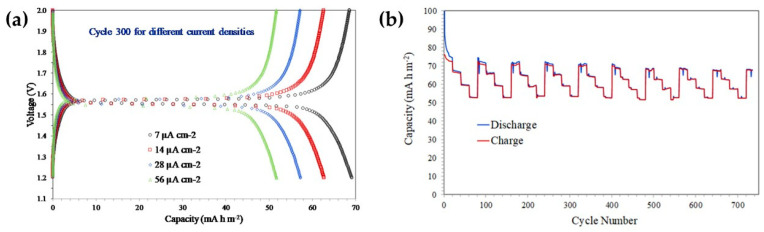
Galvanostatic discharge–charge profiles and cycling behavior of mesoporous nanocrystalline Li_4_Ti_5_O_12_ thin films heat-treated @700 °C for 15 min: (**a**) CV discharge–charge curves recorded at different current densities (7, 14, 28, and 56 cm^−2^), and (**b**) capacity as a function of cycle number during sequential rate testing. Reproduced from [41] under CC BY license.

As shown in Figure 6, mesoporous nanocrystalline Li_4_Ti_5_O_12_ thin films prepared by a sol–gel/block-copolymer route exhibit clearly improved electrochemical behavior after optimized heat treatment at 700 °C for 15 min [41]. The galvanostatic discharge–charge profiles measured at increasing current densities (7, 14, 28, and 56 A cm^−2^) show a well-defined plateau near 1.55 V, confirming the formation of the spinel Li_4_Ti_5_O_12_ phase and the preservation of the characteristic zero-strain insertion mechanism. Although a gradual reduction in capacity is observed as the current density increases, the response remains remarkably stable, indicating efficient lithium-ion transport through the interconnected porous network. In addition, the cycle-number-versus-capacity curves reveal only minor fading across successive current-density steps, demonstrating that the optimized mesostructure retains good reversibility under high-rate conditions. Overall, these results show that the combination of molecular-level sol–gel chemistry and mesostructural control is highly effective for producing LTO electrodes with improved rate capability and stable cycling behavior.

#### 2.3.2. Hydrothermal Synthesis

Hydrothermal synthesis represents an alternative wet-chemical route that enables crystallization under elevated temperature and autogenous pressure, allowing the preparation of LTO with controlled morphologies such as nanosheets, nanorods, and hierarchical architectures. Compared with solid-state synthesis, this route offers superior control over nucleation and crystal growth kinetics and can generate highly crystalline materials without the extremely high calcination temperatures usually required for diffusion-controlled ceramic processing [2,42,43].

**Figure 7:**
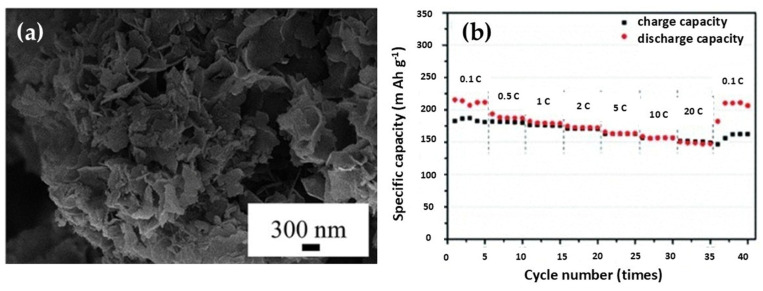
(**a**) SEM morphology of hydrothermally synthesized LTO nanosheets (2 M LiOH, 550 °C heat-treated), (**b**) rate capability 0.1 C–20 C delivering stable ~175 mAh g^−1^ capacity for 40 cycles. Note: nano-architecture enables ultrahigh-rate performance. Reproduced with permission from [43].

A representative example is the hydrothermal synthesis of LTO nanosheets reported by Wu et al., who optimized the process using Ti (OC_4_H_9_)_4_ and 2 M LiOH as precursors, followed by heat treatment at 550 °C [43]. The resulting nanosheets exhibited excellent crystallinity and stable capacity retention of 175 mAh g^−1^ across rates from 0.1 C to 20 C for 40 cycles. As shown in Figure 7, the electrochemical advantages of these hydrothermally synthesized LTO nanosheets are closely linked to their nanostructured morphology. The nanosheet architecture shortens Li^+^ diffusion pathways, increases the electrode/electrolyte interfacial area, and accommodates volume changes during cycling, facilitating rapid ion transport, enhanced electrode/electrolyte contact, and structural integrity under high-rate conditions.

Jiang et al. further demonstrated the versatility of hydrothermal synthesis by preparing hierarchical Li_4_Ti_5_O_12_/TiO_2_ composite tubes, where the controlled nanostructure improved lithium-ion transport kinetics and cycling stability [43,44]. These examples highlight the ability of hydrothermal methods to engineer morphology-specific designs that address the intrinsic limitations of bulk LTO, particularly the low electronic conductivity and sluggish Li^+^ diffusion.

However, the practical limitations of hydrothermal synthesis must also be acknowledged. The process generally relies on sealed autoclaves, extended reaction times, and batch operation, which constrain industrial scale-up despite the excellent control it offers over crystal morphology and phase purity [45,46]. As a result, hydrothermal synthesis is a powerful tool for morphology optimization, while sol–gel and solid-state routes may be more practical for large-scale production.

#### 2.3.3. Solid-State Synthesis

Solid-state synthesis remains the most industrially established and widely adopted method for LTO production due to its inherent simplicity, robustness, and scalability. In this approach, solid precursors such as Li_2_CO_3_ (or LiOH) and TiO_2_ (typically anatase) are intimately mixed and subjected to prolonged high-temperature calcination, commonly ≥800–900 °C, to enable diffusion-controlled solid-state reactions that form the spinel phase [11,36,38].

The process proceeds through several intermediate titanate phases. Shen et al. identified a pathway in which Li_2_TiO_3_ initially forms, followed by Ti^4+^ diffusion into the lattice to yield the final Li_4_Ti_5_O_12_ stoichiometry [38]. This diffusion-limited mechanism ensures reproducible phase formation but inherently favors grain growth and particle coarsening during the extended high-temperature treatment. As a result, solid-state LTO typically exhibits primary particle sizes in the sub micrometer-to-micrometer range (0.5–5 μm) with significant agglomeration, which reduces the specific surface area and lengthens Li^+^ diffusion pathways compared with wet-chemical routes [40].

Despite these microstructural limitations, solid-state synthesis offers several practical advantages that maintain its industrial relevance. The process requires no specialized equipment beyond conventional ceramic processing infrastructure, uses inexpensive and abundant precursors, and achieves high phase purity through simple stoichiometric control and extended thermal equilibration. Sun et al. emphasize that solid-state LTO provides a reliable baseline for manufacturing-scale production, where microstructural optimization must be balanced against processing cost and throughput [36]. However, these trade-offs become more evident under aqueous conditions. Coarser particle morphology and reduced surface homogeneity increase polarization during high-rate operation and limit electrolyte accessibility within the porous electrode. Prolonged high-temperature treatment can also introduce minor lattice defects or surface impurities that may accelerate interfacial reactions in water-based electrolytes. Therefore, solid-state LTO serves primarily as an economical reference material rather than an optimized choice for high-performance ALIB applications requiring nanoscale control and maximum interfacial efficiency [36,38].

The key advantage of solid-state synthesis lies in its manufacturing practicality and scalability, which outweigh microstructural limitations when production volume and cost are prioritized over peak electrochemical performance. This positions solid-state LTO as the industrial benchmark against which more sophisticated wet-chemical routes must demonstrate economically justified improvements in rate capability and cycling stability.

#### 2.3.4. Synthesis Comparison

The comparison of LTO powder synthesis routes reveals fundamental trade-offs between microstructural control and manufacturing practicality that are particularly relevant for aqueous lithium-ion battery applications. Wet-chemical approaches such as sol–gel and hydrothermal synthesis provide superior control over particle size, morphology, phase purity, and surface homogeneity, whereas solid-state synthesis excels in process simplicity and industrial scalability. These differences directly influence the resulting electrochemical performance, where nanoscale control becomes increasingly important under the interfacial limitations of aqueous electrolytes [39,40,41].

**Figure 8:**
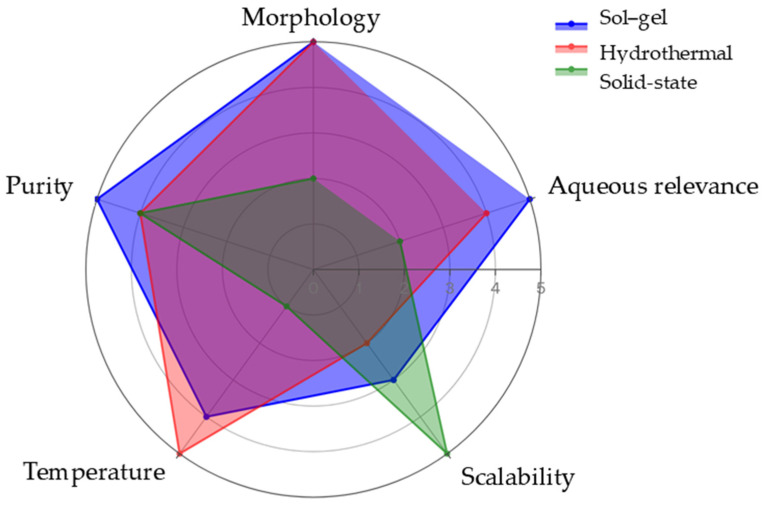
Radar chart comparing LTO synthesis routes in terms of morphology control, phase purity, calcination temperature, scalability, and relevance to aqueous electrode performance.

Figure 8 summarizes the relative strengths and limitations of the main LTO synthesis routes using a qualitative radar-chart comparison. Sol–gel synthesis offers a stronger balance between morphology control, phase purity, and relatively moderate calcination temperature, whereas hydrothermal synthesis excels in morphological engineering, but is less favorable for scale-up. Solid-state synthesis leads in scalability, but lags in microstructural optimization, making it ideal for large-scale production, but less suited for high-performance ALIB applications. This synthesis map also explains the outcomes observed in Section 2.3.1, Section 2.3.2 and Section 2.3.3. Sol–gel methods achieve nanoscale homogeneity through molecular-level precursor mixing, hydrothermal routes produce anisotropic nanostructures via confined nucleation under pressure, and solid-state processing generates robust phase formation through diffusion-controlled thermal reactions, albeit with significant grain growth. These microstructural differences directly influence electrochemical behavior of ALIB.

## 3. LTO as Anode Material for Aqueous Batteries

### 3.1. Structural Advantages

Among the various candidates explored, LTO stands out for ALIB due to moderately positive lithiation potentials (~1.55 V vs. Li^+^/Li), water inertness and minimal lattice distortion (typically <1%) [16,18] that suppress cracking and maintain electronic contact. Its near-zero volume change (~0.2%), first reported by Ohzuku et al. [14], eliminates mechanical degradation, enabling exceptional cycling stability even near the HER limit (~−1.7 V vs. Ag/AgCl or SHE in aqueous media). This robustness is particularly critical for scalable electrode fabrication methods like spray deposition and tape casting, where preserved microstructure ensures uniform immobilization and interphase stability [8,14].

To provide a clearer comparison between candidate anode materials and to highlight the rationale behind the selection of lithium titanate in ALIB research, Table 2 provides a systematic comparison of Li_4_Ti_5_O_12_ with alternative anode materials for ALIBs, highlighting the fundamental trade-off between capacity and stability that underpins the selection of LTO. High-capacity materials, particularly conversion-type oxides such as Fe_3_O_4_ and Mn_3_O_4_ (600–900 mAh g^−1^), suffer from significant drawbacks, including severe volume expansion and particle cracking during cycling, as well as catalytic promotion of the HER, ultimately leading to rapid performance degradation. Similarly, intercalation-based alternatives exhibit the following distinct limitations: graphite (372 mAh g^−1^) demonstrates instability in aqueous environments due to HER, TiO_2_ (anatase, 335 mAh g^−1^) is hindered by slow Li^+^ diffusion kinetics and surface reconstruction phenomena, while Nb_2_O_5_ (200 mAh g^−1^) is associated with increased synthetic complexity. In contrast, LTO offers a well-balanced combination of properties as follows: (a) a theoretical capacity of ~175 mAh g^−1^ alongside an exceptionally low volume change (~0.2%), characteristic of its “zero-strain” behavior. (b) High stability in aqueous electrolytes, attributed to their operating potential (~1.55 V vs. Li/Li^+^), which lies approximately 0.3 V above the HER threshold [47]. Finally, significant synthetic versatility (e.g., sol–gel and hydrothermal methods), enabling precise control over nanoscale morphology (e.g., nanosheets), as illustrated in Figure 6 and Figure 7.

Overall, the key insight is that the intrinsic structural robustness of LTO enables extensive morphological and fabrication optimization without the risk of mechanical degradation, while its relatively elevated operating potential effectively suppresses the hydrogen evolution reaction.

As illustrated, high-capacity materials suffer poor structural/interfacial stability in water (cracking, HER catalysis), while LTO delivers unmatched cycling endurance under HER-limited conditions [16]. This positions LTO as the optimal platform for electrode fabrication optimization (Section 3.2), where microstructural design amplifies inherent material advantages.

### 3.2. Fabrication and Immobilization of LTO Electrode

Electrode fabrication represents the critical step translating sol–gel LTO powder characteristics (Section 2.3) into functional anodes for ALIBs with a focus on scalable production through controlled coating uniformity, adhesion strength, and thickness optimization. Spray deposition and tape casting (doctor blade) provide complementary approaches, with their suitability primarily determined by targeted loading and manufacturability at scale, rather than by intrinsic material–performance correlations.

#### 3.2.1. Uniformity, Adhesion and Architectural Principles

In LTO anodes for ALIB, coating uniformity governs reproducible electrochemical behavior by minimizing spatial variations in ionic/electronic transport pathways that can produce localized overpotentials within water’s narrow electrochemical stability window [42,43]. Non-uniform active material distribution creates regions of elevated ionic resistance and poor electrolyte accessibility, while adhesion failure between the composite layer and current collector disrupts electronic percolation pathways essential for high-rate operation.

CMC/SBR binder systems provide adhesion (>1.5 N cm^−1^ peel strength) through hydrogen bonding with oxidized LTO/carbon black surfaces, forming mechanically robust 3D networks. However, drying-induced binder migration where polymer chains and conductive additives redistribute toward the solvent evaporation front remains a universal challenge, compromising bottom-layer adhesion and increasing interfacial impedance unless carefully mitigated [47,48,49].

**Figure 9:**
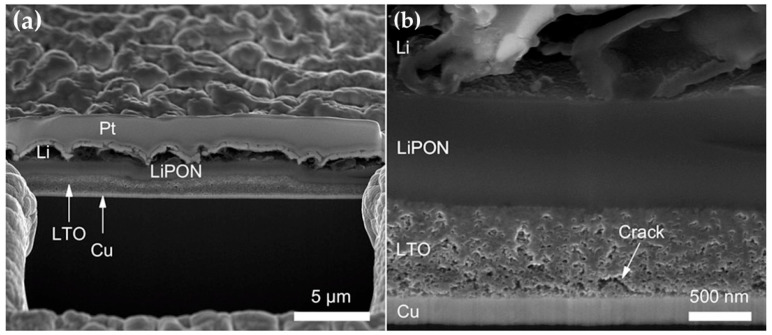
(**a**) SEM and (**b**) FIB cross-sectional images analysis of LTO layer, showing its highly porous, irregular, and comparatively thick microstructure with cracks near the copper substrate. Reproduced from [50] under CC BY license.

SEM characterization in Figure 9 provides a direct visualization of how microstructural densification governs electrode integrity in porous LTO thin films, as reported by Gockeln et al. [50]. The comparison between the more highly compressed LTO300 layer and the less-compressed LTO3.4 film reveals a clear transition from a thick, highly porous and irregular architecture toward a denser, more homogeneous and mechanically coherent structure. In the LTO300 sample, particles are more effectively rearranged into a compact network with a reduced number of voids, improved interparticle connectivity, and stronger contact to the underlying current collector, whereas the LTO3.4 film retains larger pore domains, nonuniform thickness, and crack formation in the vicinity of the Cu interface, as reported by Gockeln et al. [50] These differences are not merely morphological but functionally significant, because they directly affect charge transport, ionic accessibility, and the extent of electrochemically active material utilization. A denser and more continuous film lowers interfacial resistance, stabilizes the electron percolation network, and helps suppress local current-density hotspots that may otherwise arise from nonuniform coating development under aqueous operating conditions. At the same time, the persistence of controlled residual porosity remains advantageous, as it preserves pathways for electrolyte penetration and Li^+^ access while avoiding the severe mechanical fragility associated with overly open architectures. Thus, Figure 9 illustrates the central design trade-off in thin-film LTO electrodes: sufficient compaction is required to maximize adhesion, continuity, and electrical connectivity, but complete elimination of porosity would be counterproductive for ion transport and practical utilization. In this sense, microstructural tuning through compression acts as a practical architectural lever for balancing mechanical robustness with electrochemical accessibility in flexible thin-film battery electrodes.

Overall, the combined evidence on morphology, adhesion, and microstructural continuity shows that controlled densification of porous LTO thin films is essential for improving structural integrity and active-material utilization, while the following section examines how these architectural advantages translate into the electrochemical performance of the electrodes under operating conditions.

#### 3.2.2. Spray Deposition Technique

Spray deposition constitutes a droplet-based fabrication method uniquely suited for aqueous LTO slurries, atomizing low-viscosity dispersions (500–2000 mPa·s) into fine droplets (10–50 μm) via ultrasonic/pneumatic nozzles [48,49] and impact-heated substrates (60–120 °C) where convective airflow induces rapid solvent evaporation (milliseconds timescale), forming discrete micro-layers rather than continuous wet films characteristic of doctor blade casting [48,49].

**Figure 10:**
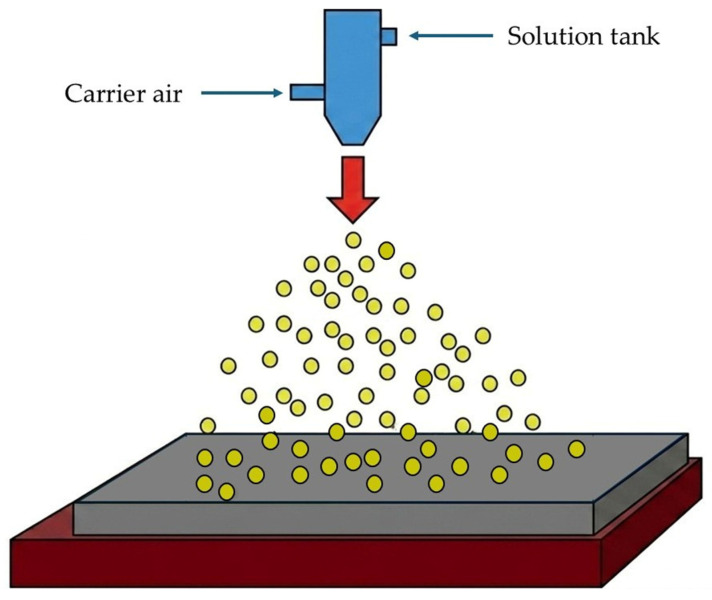
Schematic diagram of the spray coating process.

This incremental layer-by-layer mechanism eliminates shear damage to sol–gel LTO nanoparticles (Section 2.3), gravitational sedimentation during wet film leveling, and large-scale binder phase separation, while enabling precise thickness control (5–80 μm) through multi-pass deposition [49]. Critical process parameters include nozzle–substrate distance (10–20 cm governing droplet evaporation trajectory), substrate temperature (60–120 °C controlling flash drying kinetics), slurry flow rate (0.5–2 mL min^−1^ dictating deposition speed), and atomization pressure (0.5–2 bar determining droplet size distribution) [49]. Aivaliotis and Vernardou demonstrated spray-coated LTO thin films achieve ±5% thickness uniformity and superior reproducibility versus conventional processing, retaining 40–60% porosity optimal for high-rate aqueous electrolyte infiltration [49]. Figure 10 schematically illustrates this process. The coating morphology depends not only on the composition of the slurry, such as particle concentration, binder content, and viscosity, but also on process parameters including droplet size distribution, atomization pressure, substrate temperature, and evaporation kinetics.

#### 3.2.3. Tape Casting Technique

Doctor blade tape casting deposits continuous wet films of precisely controlled thickness through adjustable blade gap (30–200 μm wet), representing the industrial gold standard for high-areal-capacity electrodes compatible with roll-to-roll manufacturing [48,49]. Aqueous LTO slurries prove fully processable when viscosity exceeds 5000 mPa·s and dispersion stability optimized via pH control away from particle isoelectric point (~8–9 for TiO_2_-derived LTO), enabling mass loadings 5–20 mg cm^−2^ post-calendaring (density 2–3 g cm^−3^) attractive for stationary storage prioritizing energy over power [49,51]. CMC/SBR rheology engineering imparts shear-thinning behavior facilitating uniform spreading beneath blade while maintaining wet film integrity during transfer, though gravitational sedimentation of 100–500 nm sol–gel LTO requires dispersant optimization [47,48]. However, top-down solvent evaporation during drying generates pronounced concentration gradients driving binder and conductive additive migration toward the free surface, compromising bottom-layer adhesion to current collector and increasing interfacial impedance particularly detrimental in thick films (>100 μm) [52]. Calendaring mitigates density variations but cannot eliminate through-thickness microstructural gradients (porosity 30–50% surface vs. 20–30% collector interface) that elevate tortuosity and limit rate performance compared with the more uniform microstructure of spray-deposited electrodes [48].

These drying-induced gradients are the main limitation of tape casting in thick LTO electrodes. Although the technique delivers excellent manufacturability and high loading, its electrochemical performance depends strongly on the extent to which slurry composition, drying rate, and compaction are controlled. In practice, the method is therefore best suited to applications that value scalable production and energy density, provided that the internal heterogeneity of the coating remains manageable.

Spray deposition and tape casting should therefore be regarded as complementary rather than competing routes. Spray deposition offers greater uniformity and finer microstructural control in thin-to-moderate coatings, whereas tape casting offers superior scalability and higher mass loading [47,48,49,50,51,52,53]. The optimal choice depends on the target electrode thickness, the desired architecture, and the balance between transport efficiency and manufacturing practicality [47,48,49,50,51,52,53].

To support this qualitative comparison, Table 3 summarizes the main trade-offs between spray deposition and tape casting in terms of thickness control, uniformity, adhesion, drying sensitivity, and scalability, highlighting that the optimal choice depends on the targeted electrode architecture and application requirements.

**Figure 11:**
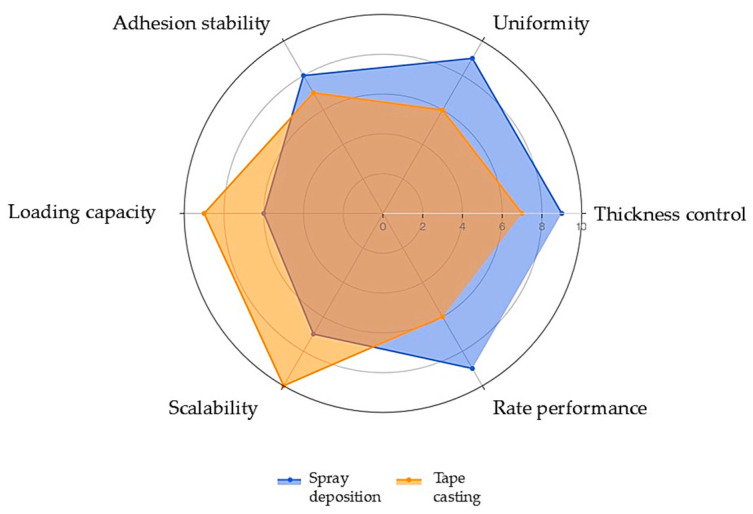
Radar chart comparing spray deposition versus tape casting performance across six LTO electrode fabrication criteria.

Figure 11 radar chart provides multi-dimensional visualization of these trade-offs, where spray deposition dominates uniformity and rate capability domains while tape casting leads in loading capacity and manufacturing scalability. The non-overlapping performance envelopes quantitatively confirm complementary positioning rather than universal superiority across all criteria.

As summarized in Table 3 and visualized in Figure 11, spray deposition offers superior lateral uniformity and microstructural control for thin-to-moderate coatings through its droplet-based mechanism, while tape casting enables higher mass loadings and industrial-scale roll-to-roll processing. Neither technique proves intrinsically superior; optimal selection depends on targeted electrode thickness, architecture requirements, and application-specific performance objectives.

This comparative evaluation underscores optimal fabrication strategy alignment with LTO’s intrinsic advantages: spray deposition maximizes nanoscale accessibility for high-rate applications, while tape casting leverages volumetric efficiency for energy-dense stationary storage.

### 3.3. Aqueous Electrolyte Engineering and Interfacial Limitations

Having established the fabrication-microstructure continuum in Section 3.2—where spray deposition produces uniform thin LTO films (5–80 μm thickness, tortuosity τ < 2) through controlled droplet-based layer-by-layer assembly that minimizes particle sedimentation and suppresses binder migration during rapid evaporation [54], versus tape casting that delivers high-areal-loading thick electrodes (30–200 μm) capable of 5–20 mg cm^−2^ despite characteristic top-down solvent evaporation gradients promoting binder segregation and increased ionic tortuosity (τ = 2.5–3.5) [55,56,57]—the present section provides detailed electrolyte engineering analysis exclusively focused on LTO-specific electrochemical performance across validated aqueous systems [11,58].

Electrolyte optimization for LTO anodes primarily targets the suppression of the hydrogen evolution reaction (HER), which occurs at potentials around ~1.55 V vs. Li/Li^+^—i.e., within the typical operating window of the material. In this context, Piffet et al. [59] demonstrated that the use of a neutral aqueous electrolyte (1 M Li_2_SO_4_, pH ≈ 6.5, ionic conductivity ~0.07 S·cm^−1^), in a three-electrode configuration (LTO as working electrode, Ag/AgCl as reference electrode, and Pt as counter electrode), enables the reversible manifestation of the Ti^4+^/Ti^3+^ redox couple. This behavior is confirmed by cyclic voltammetry (scan rate 0.5 mV·s^−1^), delivering specific capacities of approximately ~150 mAh·g^−1^ at a 1 C rate. The rationale for employing near-neutral pH conditions lies in their ability to minimize titanium dissolution, which remains below 1 ppm even after 100 cycles, thereby enhancing both the chemical stability of the electrode and its long-term cycling performance [59].

#### 3.3.1. Solvation Structure Regulation

The local coordination environment of Li ions, known as solvation structure, fundamentally governs LTO/electrolyte interfacial processes, particularly HER kinetics and interphase stability. Conventional dilute electrolytes (~1 M Li_2_SO_4_) feature primary Li^+^(H_2_O)_4–6_ solvation shells with coordination number (CN) ~4–6, facilitating proton shuttling to catalytic Ti sites and accelerating HER [12]. Water-in-salt electrolytes (>20 m LiTFSI) fundamentally reconfigure this landscape, forming anion-dominated Li^+^(TFSI)_2_ (H_2_O)_1_ shells with CN_H2O_ < 1 and H_2_O: Li^+^ molar ratio < 1 [12].

This transition reduces free water activity from 1 to 0.3 kinetically suppressing proton availability at LTO surfaces and cathodically shifting HER onset by >0.5 V (from −1.6 V to <−2.1 V vs. Li/ Li^+^). Recent studies elucidate TFSI^-^ decomposition pathways, where anion reduction generates LiF-rich passivation layers that selectively block H_2_O access while permitting Li^+^ diffusion, as confirmed by Luo et al. [60].

This solvation engineering principles complement LTO’s intrinsic zero-strain stability (Section 2.1), enabling practical ESW expansion from 1.23 V (thermodynamic limit) to >2.0 V while maintaining >99% coulombic efficiency over thousands of cycles.

#### 3.3.2. Conventional Dilute Electrolytes

Among conventional aqueous electrolytes, 1 M Li_2_SO_4_ has emerged as the established gold standard for LTO anodes, offering ionic conductivity σ = 0.05–0.08 S cm^−1^, near-neutral pH ≈ 6.5, and demonstrated chemical inertness toward both titanium current collectors and stainless-steel substrates commonly employed in LTO electrode architectures [59,61].

Its electrochemical performance has been comprehensively validated through full-cell demonstrations as follows: Placke et al. reported LiMn_2_O_4_||Li_4_Ti_5_O_12_ full cells achieving >3000 cycles at 1 C with 98% capacity retention, establishing this configuration as the baseline for aqueous LTO systems operating within the practical electrochemical stability window (ESW) of ~1.8 V [61].

Conventional dilute aqueous electrolytes (0.5–2 M), such as LiNO_3_, LiCl, and LiOH, have also been extensively explored due to their relatively high ionic conductivity (σ > 0.05 S·cm^−1^) and low viscosity, which enable efficient Li^+^ transport in three-electrode configurations (LTO working electrode on Ti foil, Ag/AgCl reference electrode, and Pt counter electrode) [54]. Early investigations focused on strong alkaline electrolytes (e.g., LiOH); however, their high pH (pH > 10) was found to induce surface passivation phenomena, thereby limiting electrochemical performance. Consequently, subsequent studies shifted toward more neutral systems such as LiNO_3_ and LiCl to improve LTO stability and reversibility [61].

Nevertheless, pH-dependent challenges remain significant. Acidic electrolytes, such as LiCl (pH ≈ 5), can accelerate titanium dissolution (>5 ppm), leading to degradation of the electrode over prolonged cycling, whereas alkaline environments promote the formation of insulating Li_2_TiO_3_ surface phases that hinder lithium intercalation kinetics [60].

**Figure 12:**
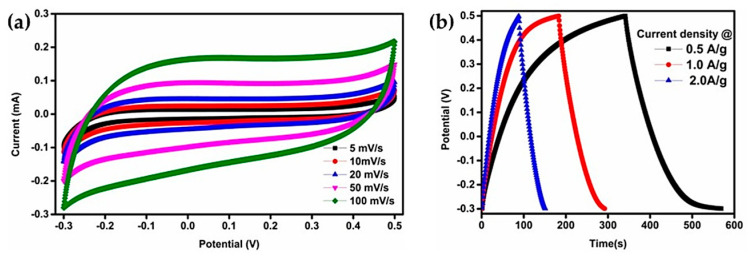
(**a**) Representative cyclic voltammograms of LTO electrode in 1 M Li_2_SO_4_ aqueous electrolyte in three-electrode configuration (LTO-working electrodeon Ti foil, Ag/AgCl reference electrode, Pt counter electrode). Scan rates: 5–100 mV/s, (**b**) Galvanostatic charge–discharge curves of LTO electrode. Reproduced from [62] licensed under CC BY-NC 4.0.

In contrast, neutral electrolytes, particularly 1 M Li_2_SO_4_ (pH ≈ 6.5, σ ≈ 0.07 S·cm^−1^), have emerged as optimal candidates, offering a balanced combination of electrochemical performance and chemical stability. As reported by Jinka et al. [62], cyclic voltammetry measurements in three-electrode cells show a quasi-rectangular response across the investigated scan-rate range, indicating predominantly capacitive behavior and good electrochemical reversibility of the LTO electrode (Figure 12a). The retention of this response from 5 to 100 mV/s suggests fast charge-storage kinetics and supports the compatibility of LTO with neutral aqueous media.

As illustrated in Figure 12a, the electrochemical response of the spinel LTO electrode remains stable within the tested potential window (~1.55 V vs. Li/Li^+^), reflecting favorable charge-storage behavior in Li_2_SO_4_-based aqueous electrolyte. Complementary galvanostatic charge–discharge (GCD) measurements (Figure 12b) further demonstrate a high specific capacity of ~160 mAh·g^−1^ at a 1 C rate, along with excellent cycling stability (~95% capacity retention after 500 cycles) and high coulombic efficiency (~99.8%).

The superior electrochemical performance of Li_2_SO_4_ electrolytes is primarily attributed to the specific adsorption of sulfate anions (SO_4_^2−^) on the LTO surface, which stabilizes the interface and effectively suppresses HER by shifting its onset potential to more negative values (~−1.8 V vs. Ag/AgCl), also discussed by Piffet et al. [59]. This interfacial effect is critical for extending the electrochemical stability window and enabling long-term operation of LTO based on ALIB systems.

#### 3.3.3. Water-in-Salt Electrolytes (WiSEs)

Water-in-salt electrolytes (WiSEs, >20 m LiTFSI) overcome the narrow electrochemical stability window of water (1.23 V thermodynamic) by saturating the aqueous medium with excess lithium bis(trifluoromethanesulfonyl)imide (LiTFSI). The resulting anion-dominated solvation shells (H_2_O:Li^+^ ratio < 1) reduce free water activity, suppressing both HER and OER, and extending the practical ESW to ~2.8–3.0 V. This enables high-voltage LTO||LMO full-cells [2,62].

Suo et al. first demonstrated this concept using 21 m LiTFSI (σ = 0.01 S/cm, viscosity ~100 cP), achieving ESW expansion validated by linear sweep voltammetry on inert electrodes [12]. For LTO anodes, WiSE performance has been systematically characterized in three-electrode configurations (LTO working electrode on Ti foil, Li metal reference electrode, and Pt wire counter electrode) [30].

**Figure 13:**
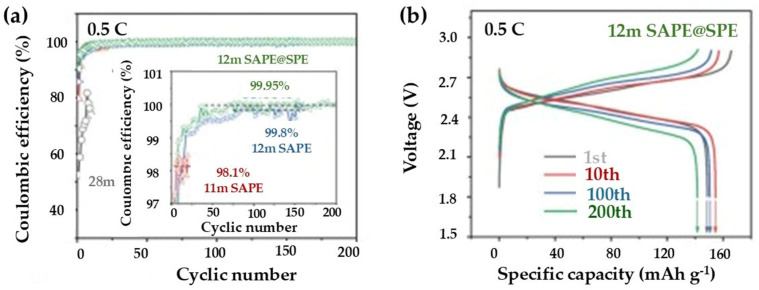
Cycling stability (**a**) of the LMO//LTO full cell in 28 M WiSE, 11 M SAPE, 12 M SAPE, and 12 M SAPE@SPE electrolytes at 0.5 C. (**b**) The voltage profiles of the 1st, 10th, 100th, and 200th cycles of the LMO//LTO full cell in 12 M SAPE@SPE electrolyte at 0.5 C (0.075 A g^−1^ for LTO, based on mass of LTO). Reproduced with permission from [30].

Figure 13 presents the electrochemical performance of the LMO//LTO full cell in various aqueous electrolytes. Figure 13a displays the long-term cycling stability of the cells in 28 M WiSE, 11 MSAPE, 12 M SAPE, and 12 M SAPE@SPE at 0.5 C. The superior capacity retention and coulombic efficiency of the 12 M SAPE@SPE electrolyte underscore the stability provided by the hybrid system. Additionally, Figure 13b shows the representative voltage profiles for the 1st, 10th, 100th, and 200th cycles in 12 M SAPE@SPE, demonstrating excellent electrochemical reversibility and structural integrity of the LTO anode during prolonged operation.

In 21 m LiTFSI (pH~4.5), LTO exhibits Ti^4+^/Ti^3+^ redox peaks at 1.55 V vs. Li/Li^+^ in CV measurements (scan rates 0.1–1 mV/s), delivering specific capacity of 145 mAhg^−1^ at 1 C with 92% capacity retention after 470 cycles (coulombic efficiency = 99.96%) [62]. Full-cell pairing LTO with Li_1_._5_Mn_2_O_4_ cathodes operates at more than 2.5 V average voltage, significantly exceeding the capabilities of conventional dilute electrolytes [62].

Despite their comparative advantages over conventional electrolytes, they present certain fundamental limitations. More specifically, the high viscosity (>100 cP) and low ionic conductivity (0.01 S/cm vs. 0.07 S/cm for 1 M Li_2_SO_4_) require thin electrodes (<50 μm thick), making WiSE particularly suitable for spray-deposited LTO membranes, as discussed in Section 3.2 [30].

Having established that WiSE enables LTO operation at extended ESW (2.8 V) through anion-dominated solvation and LiF SEI formation, hybrid electrolytes have been developed to combine the high conductivity of dilute aqueous systems with the WiSE stability. These hybrids integrate aqueous salts with ionic liquids (ILs), gel polymers (GPEs), or localized high-concentration concepts, offering tunable properties for scalable LTO electrodes.

Although WiSE has been highly effective in widening the electrochemical stability window and suppressing parasitic water reactions, their practical implementation can be constrained by the high cost of concentrated salts, increased viscosity, and the environmental burden associated with large salt loadings [63]. In this context, hybrid electrolytes provide a more balanced route because they retain the safety benefits of aqueous systems while improving conductivity, interfacial stability, and processing practicality.

#### 3.3.4. Hybrid Electrolytes

Gel-polymer electrolytes (GPEs) offer a complementary stabilization strategy by confining aqueous salts within polymer matrices, thereby limiting solvent mobility and mitigating parasitic interfacial reactions. Langevin et al. demonstrated that UV-cured gel-polymer electrolytes enable improved interfacial stability and safer operation in advanced aqueous lithium-ion configurations [64].

Localized high-concentration electrolyte (LHCE) concepts extend the concentrated-electrolyte strategy by preserving strong local salt coordination while improving transport properties relative to super concentrated systems. Shen et al. recently reported interfacial localized high-concentration aqueous electrolytes exhibiting electrochemical stability windows exceeding 3.0 V, ionic conductivities above 30 mS cm^−1^, and stable cycling performance in rechargeable ALIBs [65]. These results confirm that electrolyte composition governs interfacial chemistry rather than bulk transport alone.

Another critical parameter in aqueous electrolyte design is pH control. During cycling, local pH fluctuations arise from proton-consuming and proton-generating side reactions, particularly hydrogen and oxygen evolution reactions, as described by classical aqueous electrochemical thermodynamics [10,66]. As emphasized in comprehensive analyses of aqueous rechargeable systems by Demir-Cakan et al. [67], uncontrolled pH shifts can accelerate current collector corrosion, induce dissolution of transition-metal oxides, and destabilize electrode interfaces. Furthermore, concentrated-electrolyte studies by Yamada et al. [68] highlight that interfacial stability is strongly coupled with local proton activity and solvation structure. Near-neutral electrolytes generally provide improved stability for titanium-based anodes, whereas acidic environments favor transition-metal dissolution, and alkaline conditions accelerate hydrogen evolution kinetics [67,68,69]. Buffering strategies and careful salt selection are therefore commonly employed to mitigate pH drift and suppress voltage instability during prolonged cycling [67].

Finally, electrolyte selection must be compatible with current collectors and binder systems. As discussed by Pourbaix in classical corrosion theory [66], metal stability in aqueous media is intrinsically pH- and potential-dependent. Comparative electrolyte studies by Li et al. [45] further demonstrate that anion chemistry significantly influences corrosion behavior and electrochemical stability. Titanium, stainless steel, and carbon-based substrates exhibit superior corrosion resistance in neutral aqueous electrolytes, whereas copper and aluminum are susceptible to dissolution in chloride- or nitrate-containing media under polarized conditions [66,68]. Hydrophilic binder systems such as carboxymethyl cellulose (CMC) and styrene–butadiene rubber (SBR) are widely adopted in aqueous processing routes due to their chemical compatibility and enhanced electrolyte wettability, as broadly discussed in electrolyte and interphase reviews by Xu and co-workers [29].

In summary, advances in electrolyte engineering have significantly expanded the voltage window, safety, and interfacial stability of ALIBs. Pioneering work on water-in-salt electrolytes by Suo et al. [12], the mechanistic analyses of concentrated systems by Yamada et al. [68], and the full-cell demonstrations by Sun et al. [36] collectively illustrate how electrolyte composition governs both bulk transport and interfacial chemistry. Nevertheless, the coupling between electrolyte composition and anode interfacial reactions remains a key challenge, particularly regarding hydrogen evolution, surface instability, and degradation mechanisms that govern long-term reversibility.

While previous sections analyzed the physicochemical characteristics of individual aqueous electrolytes and electrolyte-engineering strategies, a full-cell perspective is required to assess their practical electrochemical impact. Metrics such as average voltage, specific capacity, coulombic efficiency, and cycle life emerge from the coupled interaction between electrode materials and electrolyte chemistry rather than from electrolyte properties alone [61,63].

Table 4 compiles representative literature data for hybrid organic/non-aqueous and aqueous configurations employing dilute electrolytes, water-in-salt systems, gel-polymer electrolytes, and localized high-concentration strategies [11,64], enabling quantitative evaluation of how electrolyte design governs voltage window, interfacial stability, and long-term performance across LIB architectures.

Hybrid organic/non-aqueous based on LIBs with carbonate electrolytes operate at high average voltages of approximately 3.7–3.8 V and deliver superior gravimetric energy densities [3,4,8], but carry flammable solvents and increased system complexity [4].

In contrast, ALIBs employing neutral or mildly concentrated electrolytes (e.g., Li_2_SO_4_ or LiNO_3_) exhibit lower operating voltages in the range of 1.8–1.9 V, yet demonstrate remarkable cycling stability and high coulombic efficiency, as reviewed by Wang et al. and Demir-Cakan et al. [57,67], making them well-suited for stationary and grid-level applications, where safety and durability outweigh maximum energy density requirements.

Importantly, pioneering work by Suo et al. on water-in-salt electrolytes [12], further analyzed mechanistically by Yamada et al. [68] and experimentally validated in full cells by Sun et al. [36], demonstrated that highly concentrated electrolytes can expand the practical electrochemical stability window of water to nearly 3.0 V. Such electrolyte-engineering strategies significantly narrow the performance gap between aqueous and non-aqueous systems while maintaining intrinsic non-flammability and improved safety [65].

Overall, Table 4 supports the conclusion that ALIBs constitute a competitive alternative for applications prioritizing safety, durability, and sustainability, particularly when electrolyte design is carefully optimized to regulate interfacial chemistry and suppress parasitic reactions [19,60,61].

**Table 4:** Comparison of electrolyte engineering strategies in LIBs.

Electrolyte Type	Concentration	Representative System	Set Up	Capacity	Electrochemical Window (V)	Cyclic Retention	Limitations	Ref.
Dilute aqueous	1–2 M (Li_2_SO_4_, LiNO_3_)	LTO|LiMn_2_O_4_	3-electrode system (WE, RE: Li, CE: Pt)	110	1.2–1.4	5000	HER Low voltage	[60,61]
WiSE	21 m (LiTFSI)	LTO|Li_1.5_Mn_2_O_4_	3 electrodes	145	2.8	470	High viscosity Cost	[65]
GPE	PVA-Li_2_SO_4_	LTO|LiFePO_4_	Full cell	115	1.8–2.0	3000	Lower conductivity	[60]
LHCE	LiTFSI + sulfolane	LTO|LiMn_2_O_4_	Full cell	132	2.5	800	Complex chemistry	[61]
Polymer-WiSE	12 m LiTFSI + PEO	LTO|LMO	3 electrode	142	3.86	200	Complex chemistry	[65]

## 4. Discussion

### 4.1. Summary of Key Findings

The literature reviewed highlights growing scientific interest in ALIBs as safe and environmentally benign alternatives to conventional non-aqueous lithium-ion systems. Their appeal lies in the intrinsic safety, low cost, and non-flammability of aqueous electrolytes, making them particularly attractive for stationary and large-scale energy-storage applications. However, the narrow thermodynamic stability window of water and parasitic interfacial reactions remain key limitations.

The electrochemical performance of LTO is strongly influenced by synthesis conditions and resulting microstructural characteristics. Particle size, phase purity, crystallinity, and surface chemistry all play critical roles in determining lithium diffusion kinetics, electronic conductivity, and interfacial stability. Wet-chemical synthesis routes such as sol–gel and hydrothermal methods offer enhanced control over these parameters compared with conventional solid-state synthesis, enabling nanoscale particles with improved electrochemical accessibility, albeit with trade-offs between microstructural control, processing complexity, and scalability.

In addition to material synthesis, electrode fabrication and immobilization strategies represent a crucial step in translating intrinsic material properties into practical electrochemical performance. Architecture, including thickness, porosity, uniformity, and adhesion, governs ion and electron transport, while inadequate microstructure can limit the benefits of even high-quality powders.

### 4.2. Electrode Fabrication: Trade-Offs and Scalability

The comparative analysis of spray deposition and tape casting highlights the fundamental role of fabrication strategy in controlling electrode morphology and performance. Spray deposition produces electrode layers through incremental droplet deposition and rapid solvent evaporation, inherently suppressing large-scale particle sedimentation and reducing binder migration during drying—resulting in highly uniform coatings that are particularly beneficial in aqueous systems where localized current-density heterogeneities can accelerate parasitic reactions. However, spray deposition requires stable dispersions and is highly sensitive to parameters such as spray rate, atomization pressure, substrate temperature, and nozzle–substrate distance; at higher loadings, multilayer buildup may introduce transport limitations unless porosity and architecture are carefully engineered.

Tape casting, in contrast, remains the most widely used electrode fabrication method in industrial lithium-ion battery manufacturing, enabling thick electrodes with high mass loading (30–200 μm, 5–20 mg cm^−2^) essential for achieving high areal capacity. Nevertheless, thick tape-cast electrodes frequently develop concentration gradients in binder distribution, conductive-additive content, and porosity during drying, increasing ionic tortuosity and electronic resistance, effects particularly pronounced in aqueous electrolytes.

These obsevations demonstrate that spray deposition and tape casting should not be viewed as competing techniques but rather as complementary fabrication strategies. Spray deposition offers superior control over coating uniformity and microstructural homogeneity in thin or moderately thick electrodes, whereas tape casting provides clear advantages in terms of scalability and high mass loading. The choice of fabrication method therefore depends on the targeted electrode architecture and application requirements.

### 4.3. Material–Electrolyte–Architecture Synergies

A central conclusion emerging from this work is that electrochemical performance in ALIBs cannot be attributed to a single material parameter. Instead, performance arises from the combined interaction among active material properties, electrolyte chemistry, and electrode architecture. LTO provides excellent structural stability and a relatively favorable working potential for aqueous environments, yet these intrinsic advantages alone are insufficient to guarantee stable battery operation. Effective electrodes must provide efficient transport pathways for both lithium ions and electrons, maintain mechanical integrity, and sustain stable interfaces with the electrolyte. At the same time, electrolyte composition influences interfacial reactions, pH stability, and parasitic gas evolution processes that directly affect electrode stability.

Consequently, the design of high-performance aqueous lithium-ion batteries requires a holistic approach that simultaneously considers material synthesis, electrode fabrication, and electrolyte selection. Improvements in only one component of the system are unlikely to produce substantial performance gains if the remaining elements remain poorly optimized.

### 4.4. Persistent Research Gaps

Despite the significant advances, several challenges remain in the development of LTO-based aqueous battery systems. First, the impact of electrode fabrication parameters on electrochemical performance, particularly for spray-based deposition techniques, is not fully understood. While studies have demonstrated improved uniformity in spray-coated electrodes, systematic investigations linking process parameters to resulting electrode microstructure and transport properties remain limited. Second, most studies investigate either material synthesis or electrode fabrication independently, without considering the combined effects of these processes on final device performance. Integrating synthesis methods, particle morphology, electrode architecture, and electrolyte chemistry could provide valuable insight into performance optimization strategies. Finally, the majority of studies focus on half-cell configurations rather than full aqueous battery systems. Advancing stable aqueous full cells with compatible cathodes and optimized electrolytes remains a critical step toward practical implementation. Collectively, these gaps suggest the need for system-level optimization rather than isolated improvements in individual components.

## 5. Perspectives

### 5.1. Recommended Research Directions

Future experimental work could therefore focus on establishing a controlled experimental matrix in which synthesis method, electrode architecture, and electrolyte composition are varied simultaneously. Such an approach would enable a more comprehensive understanding of how microstructural characteristics of LTO particles interact with electrode morphology and electrolyte chemistry to determine electrochemical performance.

Spray-deposited electrodes, in particular, merit detailed investigation under well-controlled fabrication conditions. While spray deposition offers clear benefits in coating uniformity and reduced binder migration, its polyparametric nature means that small changes in process parameters can significantly influence electrode microstructure. Systematic studies of parameters such as spray rate, substrate temperature, and droplet size distribution could elucidate their impact on porosity, adhesion, and electrochemical behavior, ultimately enabling reproducible processing windows for scalable electrodes in ALIBs.

### 5.2. Exploration of Advanced Electrolyte Concepts

Future research should explore the compatibility of LTO anodes with various high-concentration electrolyte formulations, with particular attention to interfacial stability and long-term cycling behavior. Studies examining how these electrolytes influence solid–electrolyte interphase formation, gas evolution kinetics, and electrode corrosion would provide valuable insight into the mechanisms that govern stability in aqueous battery systems.

### 5.3. Toward LTO/LMO Aqueous Full Cells

While many studies focus on half-cell configurations to evaluate anode performance, the ultimate objective of aqueous battery research is the development of stable full-cell systems capable of practical energy storage. One of the most promising cathode candidates for pairing with LTO anodes in aqueous environments is lithium manganese oxide (LiMn_2_O_4_, LMO). The relatively high operating potential of LMO and its compatibility with neutral aqueous electrolytes make it an attractive counterpart for constructing aqueous lithium-ion full cells.

Future work could therefore investigate the design and optimization of LTO/LMO aqueous full-cell architecture. Key parameters to be explored include electrode balancing, electrolyte composition, and electrode thickness ratios that maximize energy efficiency while maintaining long-term stability. Attention should be given to minimizing polarization losses and suppressing parasitic reactions at both electrodes. By integrating optimized synthesis routes, advanced electrode fabrication strategies, and carefully engineered electrolyte systems, it may be possible to achieve aqueous battery systems that combine safety, stability, and competitive electrochemical performance.

### 5.4. Outlook

The future advancement of ALIBs will require the integrated optimization of material synthesis, electrode architecture, and electrolyte chemistry rather than incremental improvements of individual components. LTO-based electrodes represent a promising platform for investigating the interplay between structural stability, interfacial chemistry, and electrode fabrication techniques in aqueous environments. Continued research along these directions could lead to battery systems that retain the intrinsic safety and environmental benefits of aqueous electrolytes with performance levels suitable for large-scale energy storage. Key priorities include validating LTO/LiMn_2_O_4_ full-cells, demonstrating roll-to-roll spray–tape hybrid manufacturing, and deploying pilot systems for grid-scale applications.

## 6. Conclusions

This review highlights lithium titanate as a structurally robust and intrinsically safe anode material for aqueous lithium-ion batteries, emphasizing how synthesis route, electrode fabrication, and electrolyte design collectively govern its electrochemical performance. Wet-chemical synthesis methods enable nanoscales, phase-pure LTO powders, while scalable fabrication strategies such as spray deposition and tape casting translate these materials into electrodes with tunable architecture and transport properties. Complementary advances in electrolyte design, including highly concentrated and hybrid aqueous systems, offer promising pathways to mitigate parasitic reactions and extend the practical operating window of LTO-based cells. The combined optimization of LTO synthesis, engineered electrode structures, and tailored aqueous electrolytes provides a credible route toward safe, durable, and industrially relevant aqueous battery technologies for stationary and large-scale energy storage applications.

## Figures and Tables

**Table 1:** Comparison of representative organic and aqueous LIB systems.

System/ Electrodes	Electrolyte	Average Cell Voltage (V)	Specific Capacity (mAh g^−1^)	Cycle Life	Coulombic Efficiency	Features	Ref.
LiFePO_4_-LTO	1 M LiPF_6_ in EC/DMC (1:1)	~1.85	130	>1200	>99	Flat voltage Flammable organic electrolyte	[3]
CNTs/LTO-LiFePO_4_	1 M LiPF_6_ in EC/EMC	2.5	110 @10 C	>1500	>99	High capacity retention	[8]
LiMn_2_O_4_-LTO	1 M Li_2_SO_4_ (aq.)	~1.8	110	>5000	~99	High stability	[11]
						Safe operation	
LiFePO_4_-LTO	2 M LiNO_3_ (aq.)	~1.9	120	>3000	~98	Excellent reversibility	[11]
LiFePO_4_-Li_4_Ti_5_O_12_	PVA–Li_2_SO_4_ gel	1.8	115	3000	~98	Flexible polymer system	[11]

**Table 2:** Comparative overview of representative anode materials investigated for ALIBs, highlighting the trade-off between theoretical capacity and electrochemical stability in water.

Anode Material	Charge Storage Mechanism	Theoretical Specific Capacity (mAhg^−1^)	Volume Change	Limitations in Water	Ref.
Graphite	Li^+^ intercalation	372	~10%	Strong HER Unstable potential data	[22,45]
TiO_2_ (anatase)	Intercalation	~335	~4%	Slow diffusion Surface reconstruction	[21,22,25,26]
TiO_2_ (brookite)	Intercalation	~300	~3.5%	Defect-incluced HER activation	[22,26]
LiTi_2_(PO_4_)_3_ (NASICON)	Intercalation	~170	~1%	pH sensitivity Dissolution risk	[22,45]
Nb_2_O_5_	Pseudocapasitive/intercalation	~200	~2%	Complex synthesis Stability issues	[21]
Conversion type oxides (Fe_3_O_4_, Mn_3_O_4_)	Conversion reactions	600–900	~50%	Large volume change Rapid degradation	[22,35]
Li_4_Ti_5_O_12_	Intercalation (Ti^4+^/Ti^3+^)	175	~0.2%	Low electronic conductivity	[14,19,20,21]

**Table 3:** Comparative summary of spray deposition and tape casting techniques for LTO-based aqueous anodes, highlighting trade-offs in thickness control, uniformity, adhesion, formulation sensitivity and scalability.

Criterion	Spray Deposition	Tape Casting	Ref.
Typical thickness regime	Thin-moderate coatings (5–80 μm) Multi-pass control	Moderate-thick coatings (30–200 μm) Blade gap control	[47,48,49,50]
Coating uniformity	High lateral uniformity minimal sedimentation	Sensitive to rheology/drying gradients	[48,51]
Adhesion to collector	Strong via rapid droplet drying	Migration risk increases with thickness	[48,52,53]
Drying dynamics	Low binder migration risk	High sensitivity to top-down segregation	[51,52,53]
Scalability	Research/optimized R2R	Industrial R2R established	[51,52,53]

## Data Availability

No new data were created or analyzed in this study.
